# Nutriment

**Published:** 1883-11

**Authors:** 


### NUTRIMENT.

ANY derangement of the machinery of life, whether of mental or physical origin, is liable to be followed by waste of the tissues of the body, and as a consequence a loss of health and strength. Without stopping to give reasons for a fact that is within the experience or observation of every one, we venture to say that the most rational remedy for waste is a new supply of the same kind of material wasted: and moreover that this restorative shall be introduced into the system by the most direct and speedy means, and with the least loss of its nutrient elements.

There can be no increase of strength except through increase in the quantity and improvement in the quality of the blood. In extreme cases transfusion has been employed, but in ordinary cases it is a plan fraught with too much danger and too many inconveniences to be resorted to. Pure blood is, of course, the material which will most readily and most directly supply what is required by a system impoverished from its loss. But there are three conditions on which the success of this treatment depends.

FIRST—That a fresh supply shall be attainable at all times.

SECOND—That it shall be administered with the least possible inconvenience to the patient.

THIRD—That it shall reach the circulation in its purity and as speedily and directly as possible.

The inventive genius of Americans, that tries its skill in every department of human industry, has not forgotten the invalid, and fortunately these difficulties have recently been swept away.

PARKE, DAVIS & Co., manufacturing chemists, whose works are at Detroit, Mich.—but whose business extends over the whole continent, and even to other continents—are manufacturing DESSICATED BULLOCK'S BLOOD that seems to fulfill the first condition of a constant supply of fresh blood most admirably.

The other conditions are amply met by using as vehicles to introduce blood or any other nutriment into the system of the well known HOLLOW SUPPOSITORIES, to be found at all druggists. These hollow suppositories are of several sizes, and will hold from half a drachm to half an ounce of desiccated blood. For adults we would advise the use of size C, although size 3 is probably more frequently used for nutrients. If a hollow suppository is filled with desiccated blood and inserted according to directions, the blood and the suppository itself—which is made of the purest butter of cacao—will be absorbed in half the time required by the digestive organs when taken into the stomach, besides avoiding the obvious disadvantages of having its value impaired by mingling with the contents of the stomach.

We venture to predict that in the near future desiccated blood will occupy the first place in the list of nutrient substances, and that hollow suppositories will be exclusively used as vehicles to introduce it into the system.

